# Myocardial Infarction and Complete Heart Block in a Patient With Anomalous Origin of the Left Coronary Artery From the Pulmonary Artery (ALCAPA)

**DOI:** 10.7759/cureus.62016

**Published:** 2024-06-09

**Authors:** Muhammad Umer Riaz Gondal, Devi Parvathy Jyothi Ramachandran Nair, Smit Shah, Muhammad Zafar, Muhammad Asad Hanif, Brian McCauley, Earl Hope

**Affiliations:** 1 Internal Medicine, Reading Hospital, West Reading, USA; 2 Internal Medicine, Tower Health Medical Group, West Reading, USA; 3 Cardiology, Reading Hospital/Tower Health, West Reading, USA; 4 Cardiology, Reading Hospital, West Reading, USA

**Keywords:** anomalous origin of the left coronary artery from the pulmonary artery (alcapa), surgical correction, congenital anomaly, complete heart block, non-st segment elevation myocardial infarction (nstemi)

## Abstract

Anomalous origin of the left coronary artery from the pulmonary artery (ALCAPA) is a rare congenital malformation. We present a case of an elderly patient with ALCAPA presenting with complete heart block and non-ST-elevation myocardial infarction years after diagnosis and surgical correction. An 81-year-old female with a history of ALCAPA presented to the emergency department with chest pain and progressive mental deterioration. She was bradycardic and hypotensive. An electrocardiogram revealed a complete heart block. Troponin was 4.04 ng/mL. She received atropine and underwent transcutaneous pacing. Left heart catheterization revealed complete occlusion of the mid-left circumflex artery, which was intervened with balloon angioplasty and chronic total occlusion of the right coronary artery. She was supported with temporary transvenous pacing, did not require further pacing support, and was discharged home. Previous records unearthed that in 1988 she had presented with syncope and was diagnosed with ALCAPA, filling from right-to-left collaterals with large and ectatic coronaries. At the time, she underwent surgical correction with excision of the left coronary from the pulmonary artery and reimplantation in the left coronary cusp along the posterior aorta. She had remained asymptomatic after her surgery until this presentation. ALCAPA is extremely rare in adults. Insufficient collaterals to the left ventricle cause inadequate blood supply, leading to ischemia in adults, predisposing them to arrhythmias and risk of sudden death. Adults with ALCAPA remain at increased risk of adverse cardiac events later in life, requiring long-term monitoring.

## Introduction

Anomalous origin of the left coronary artery from the pulmonary artery (ALCAPA), also known as Bland-White-Garland syndrome, is a rare childhood congenital cardiac malformation with a high infant mortality rate. It accounts for 0.25-0.5% of all congenital heart disorders, with the incidence reported as 1/3,00,000 [1]. The congenital anomaly carries a high risk of infant mortality, with up to 90% reported in the first year of life without surgical correction [2]. Elderly survivors are rare, with our patient presenting with complete heart block and non-ST-elevation myocardial infarction years after diagnosis and surgical correction.

This article was previously presented as a meeting abstract at the 2024 American College of Cardiology (ACC) meeting on April 6th, 2024.

## Case presentation

An 81-year-old female with persistent atrial fibrillation and ALCAPA presented to the emergency department with chest pain. Chest pain was substernal, crushing, and severe, with no radiation. Her previous medical records revealed that in 1988 she had presented with syncope and ventricular ectopy and was diagnosed with ALCAPA after an urgent catheterization, filling from right-to-left collaterals with large and ectatic coronaries. At the time, she underwent surgical correction with excision of the left coronary from the pulmonary artery and reimplantation in the left coronary cusp along the posterior aorta. The patient had remained asymptomatic from a cardiovascular standpoint until the age of 45 years when she was first diagnosed with ALCAPA. After her surgical correction, she led a regular, healthy life with close follow-ups until this recent presentation.

**Figure 1:**
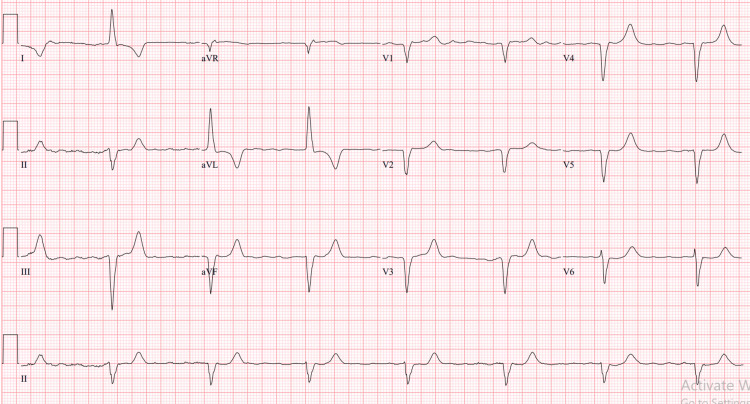
EKG showing complete heart block.

In the emergency room, vitals included a heart rate of 42 bpm and blood pressure of 86/40 mmHg. Her mental status progressively deteriorated. An electrocardiogram revealed a complete heart block (CHB) (Figure 1).

**Figure 2:**
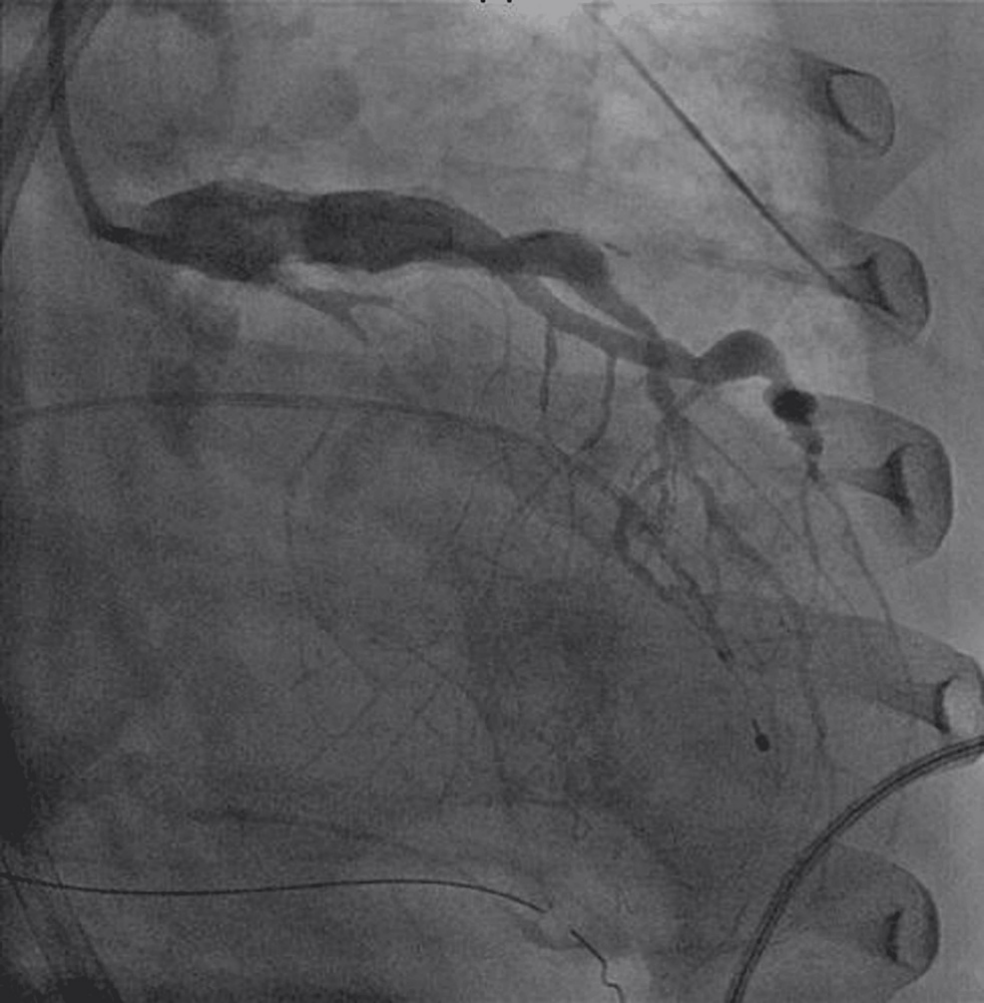
Left heart catheterization, right anterior oblique caudal view showing 100% occluded left circumflex artery.

**Figure 3:**
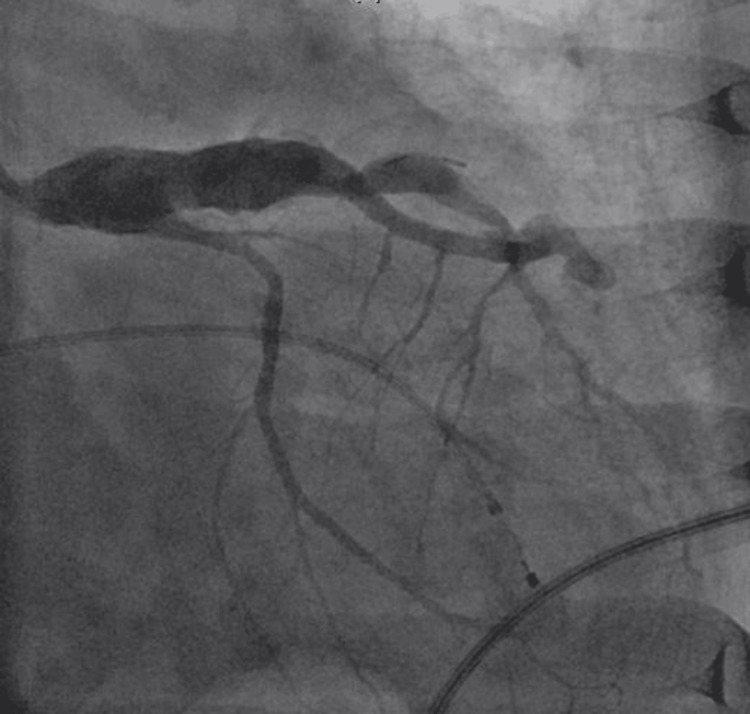
Left heart catheterization, right anterior oblique caudal view showing revascularized atretic left circumflex artery.

Troponin was high at 4.04 ng/mL. Her blood work was unremarkable, including infectious workup and thyroid stimulating hormone (TSH) (Table 1). The patient received two doses of atropine and transcutaneous pacing for her CHB. An emergent left heart catheterization revealed a calcified and aneurysmal left anterior descending artery, with a complete occlusion of the mid-left circumflex artery. It also showed chronic total occlusion of the right coronary artery (RCA). She was supported with temporary transvenous pacing. Percutaneous balloon angioplasty was performed on the left circumflex artery, and her CHB was deemed likely due to transient ischemia of the AV node from the occlusion of the left circumflex artery (providing collaterals to the RCA) (Figures 2, 3). She did not require further pacing support and was discharged home with optimal acute coronary syndrome (ACS) management.

**Table 1:** Blood work revealing elevated troponin 4.04 (ng/mL). All other lab work was within normal limits.

Variable	Labs (on admission)	Reference range
White blood cells (cells/mm^3^)	6100	4.5-11.0
Hemoglobin (g/dL)	12.9	12.0-16.0
Creatinine (mg/dL)	1.0	0.5-1.1
Alanine aminotransferase (IU/L)	16	10.0-40.0
Aspartate aminotransferase (IU/L)	38	10.0-40.0
Troponin I (ng/mL)	4.04	<0.06
B-type natriuretic peptide (pg/mL)	81	0-100
Lactic acid (mmol/L)	0.7	0.6-1.4

## Discussion

In ALCAPA, the left coronary artery arises from the pulmonary artery and carries deoxygenated blood to the left side of the heart. ALCAPA is classically divided into two types, infantile and adult-onset, based on the age of onset and symptom presentation [3]. The amount of collateral circulation between the RCA and LCA determines the extent of ischemia and symptoms. The hallmark of the adult form of ALCAPA is the significant collateral circulation between the RCA and LCA and retrograde perfusion of the left ventricle through the RCA [4]. However, over time, these collaterals are often insufficient to supply the left ventricle adequately, which can lead to subendocardial ischemia. Thus, they are at an increased risk of ventricular arrhythmia. Adults can, therefore, present with sudden cardiac death.

Approximately 90% of patients have sudden cardiac death at the age of 35 years [5]. Adult patients can remain asymptomatic or present with heart failure, angina on exertion, and mitral regurgitation. Only 10-15% of patients survive to adulthood [6]. Over the age of 50 years, survival is rare [7]. A literature review by Yau et al., involving 153 cases, revealed the average reported age of ALCAPA patients was 41 years, with the oldest being 83 years. While ventricular arrhythmias, syncope, or sudden death were observed in 17% of these patients, angina, dyspnea, or palpitations were the presenting symptoms in 66% of them, and 14% of the patients were asymptomatic [1]. There were a few case reports of ALCAPA patients presenting in their 70s [8,9]. We did not encounter any patient in their 80s with ALCAPA in our literature search.

Our patient was diagnosed with ALCAPA at the age of 46 years, as she had presented with syncope and ventricular ectopy. Before the advent of advanced radiographic technology, ALCAPA was usually diagnosed with coronary artery angiography, which is the gold standard for diagnosis [10]. The primary modalities include 2D transthoracic echocardiography and color Doppler flow imaging. Other imaging modalities that provide direct visualization of coronary artery anatomy with 3D reconstruction are CTA and cardiac magnetic resonance (CMR).

The prevalence of adult individuals with ALCAPA syndrome has significantly increased as a result of recent developments in non-invasive cardiac imaging [1]. According to the 2018 American Heart Association/American College of Cardiology and 2020 European Society of Cardiology guidelines, once ALCAPA is diagnosed, surgical correction is recommended to restore double coronary circulation [11]. Early surgical correction is recommended in adults, given that they are at increased risk of sudden cardiac death. A study by Kanoh et al. comparing long-term postoperative outcomes of ALCAPA patients revealed that adult-type ALCAPA patients had a higher risk of major adverse cardiac events, including arrhythmias and cardiac death compared to infant-type ALCAPA because of irreversible left ventricular remodeling caused by long-term chronic myocardial ischemia [12].

## Conclusions

ALCAPA is a rare congenital cardiac anomaly, and early diagnosis is essential for long-term survival. Postoperative ALCAPA patients require long-term follow-up as chronic myocardial damage can lead to adverse consequences, such as our patient who presented with non-ST-elevation myocardial infarction (NSTEMI) and complete heart block. Our case raises awareness of the association of ALCAPA with acute coronary syndrome and complete heart block.
